# Genetic predispositions of Parkinson’s disease revealed in patient-derived brain cells

**DOI:** 10.1038/s41531-020-0110-8

**Published:** 2020-04-24

**Authors:** Jenne Tran, Helena Anastacio, Cedric Bardy

**Affiliations:** 1 Laboratory for Human Neurophysiology and Genetics, South Australian Health and Medical Research Institute (SAHMRI), Adelaide, SA Australia; 2 College of Medicine and Public Health, Flinders University, Adelaide, SA Australia

**Keywords:** Induced pluripotent stem cells, Cellular neuroscience

## Abstract

Parkinson’s disease (PD) is the second most prevalent neurological disorder and has been the focus of intense investigations to understand its etiology and progression, but it still lacks a cure. Modeling diseases of the central nervous system in vitro with human induced pluripotent stem cells (hiPSC) is still in its infancy but has the potential to expedite the discovery and validation of new treatments. Here, we discuss the interplay between genetic predispositions and midbrain neuronal impairments in people living with PD. We first summarize the prevalence of causal Parkinson’s genes and risk factors reported in 74 epidemiological and genomic studies. We then present a meta-analysis of 385 hiPSC-derived neuronal lines from 67 recent independent original research articles, which point towards specific impairments in neurons from Parkinson’s patients, within the context of genetic predispositions. Despite the heterogeneous nature of the disease, current iPSC models reveal converging molecular pathways underlying neurodegeneration in a range of familial and sporadic forms of Parkinson’s disease. Altogether, consolidating our understanding of robust cellular phenotypes across genetic cohorts of Parkinson’s patients may guide future personalized drug screens in preclinical research.

## Introduction

Parkinson’s Disease (PD) is a complex neurological disease, affecting approximately 2% of the population over 60 years of age. Since the first reports of PD correlation with the *SNCA* gene^1–5^, an increasing number of genomic predispositions has been identified as direct contributors or risk factors for the disease^6–15^. In the last decade, advances in cellular reprogramming and induced pluripotent stem cell (iPSC) technology have led to the development of patient-derived brain tissue engineering^16–18^. The culturing of live patient-derived neurons provides a unique opportunity to study the cellular mechanisms of genetically-linked diseases in vitro^19,20^. IPSC studies of brain disorders remain laborious and expensive, which limits the number of cell lines that can be studied in a single laboratory and may raise the issue of statistical power and reproducibility. The field is still in its infancy, and neuronal differentiation protocols are continuously improved upon, which makes technical harmonization between studies challenging^21^. Like any model, iPSC studies have their weaknesses, and current limitations are being addressed collectively by the research community^22,23^. Nevertheless, the urgent need for better treatments for brain disorders justifies the requirement for pioneering iPSC studies modeling those diseases in vitro now. Since 2011, more than 385 neuronal lines from PD patients (and control subjects) have been generated across 67 original independent studies^24–92^. Independent phenotypic characterization of these lines revealed known and novel impairments in a range of cellular functions associated with PD. Moving forward, it is essential to integrate and compare the results obtained from these studies to identify the most reliable disease neuronal phenotypes before expanding the work to large drug screens. The identification of robust phenotypes of neurons derived from Parkinson’s patients’ iPSCs may provide the basis for a new paradigm in preclinical drug development and accelerates the progression towards clinical trials.

To quantify the prevalence and penetrance of genes known to be associated with PD, we analyzed 50 epidemiological^93–129^ and 24 genomic studies^8,10–14,130–148^ and a genome-wide association studies (GWAS) database^149^. Within this context, we then examined recent findings from human induced pluripotent stem cell (hiPSC) models that shed light on the interplay between genetic predispositions and brain cell phenotypes in people living with PD. We present an analysis of 385 human iPSC-derived neuronal lines from 67 studies, which point towards specific impairments of dopaminergic neurons from people living with PD. In comparison with the prevalence of causal PD genes in epidemiological studies, our analysis underlines the current bias of PD iPSC studies towards a subset of familial genetic predispositions. We also summarize the methodological overlap and differences between these studies, which use a broad range of reprogramming methods. Finally, our meta-analysis highlights the possibility of converging molecular and cellular pathways underlying neurodegeneration in familial and sporadic PD with diverse genetic predispositions. In particular, we discuss the convergence of various genetic predispositions on the impairment of cellular pathways underlying metabolic function, synaptic communication, inflammation, and the recycling of damaged protein and organelles. The collective insights from independent iPSC disease studies, which are pioneering this blooming field of preclinical research, may guide us towards translating fundamental discoveries into effective treatments for patients.

## The interplay between genomic predispositions and environmental factors leads to Parkinson’s

**Fig. 1:**
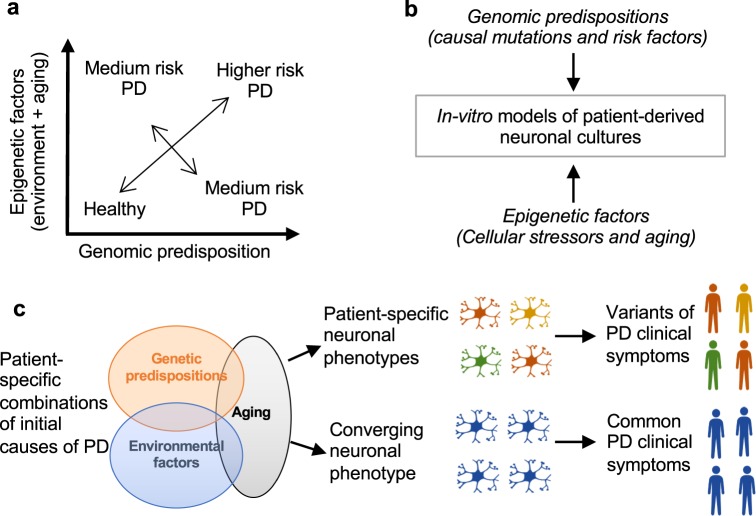
A combinatorial spectrum of genetic risks, cellular stressors, and brain cell dysfunctions causes Parkinson’s disease. **a** Graphical overview of PD risk associated with genomic predispositions and epigenetic factors. **b** Schematic overview of PD etiological trajectories in iPSC models. **c** Unique cellular phenotypes may cause PD symptoms in a subset of patients (individualized etiology) and the convergence of various initial causes into common cellular phenotypes may cause other symptoms (convergent etiology).

In the mid-1990s, the connection between PD and underlying genetic mutations was established^4,5,150^. It is now evident that varying degrees of the interplay between genomic predispositions and aging and cellular stressors impose a risk for disease^151^ (Fig. 1a). Previous studies have shown vascular insults to the brain, repeated head trauma, neuroleptic drugs, exposure to pesticides, and manganese toxicity increase the risks of developing symptoms of PD^152–154^. In addition, advancing age can also cause a cascade of stressors within the substantia nigra, which weakens the neurons and their ability to respond to further insults^155,156^. Ultimately, the uniqueness of the interactions between genes and the environment makes the development of a single treatment for PD difficult as they give rise to a spectrum of neuronal phenotypes that can be unique to individual patients (Fig. 1c). The development of a model with the ability to replicate the genomic and epigenetic aspects of the disease is crucial (Fig. 1b). As increasing evidence suggests that genetic mutations are key modulators of disease initiation and progression, the identification and understanding of the various genomic predispositions are required for the development of better-targeted treatments to slow the disease progression.

## Learning from genetic analyses of PD case–control studies

**Fig. 2:**
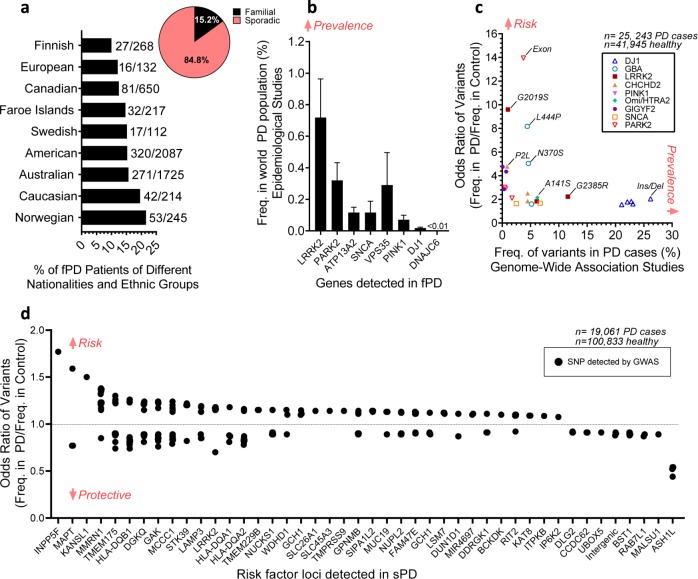
The genomics of Parkinson’s disease: prevalence and penetrance. **a** In the world-wide population of people living with PD, ~85% of PD cases are sporadic (sPD) and the remaining are familial (fPD) (*n* = 5650 PD cases combined, refer to “Methods”). **b** Genetic mutations occur at low (< 1%) and varying frequencies (Freq.) in the PD world population (*n*=488 patients carrying mutation, 32,012 total PD cases used for analysis, refer to “Methods”). Data represented as the mean±SEM. **c** GWAS data suggests risk variants (OR>1.5) in fPD genes tend to be less prevalent in PD cases (*n* = 25,243 PD cases, 41,945 healthy, refer to “Methods”). **d** Single nucleotide polymorphisms (SNPs) in over 44 genomic regions show significant (*p* < 5 x 10^−8^) association to PD. Each point presents an independent SNP hit associated with PD.

We analyzed the reports from 12 international studies^94,157–167^, totaling 5650 persons living with PD in North America, Europe, and Australia. We confirmed that globally only 15% of patients report a family history of PD symptoms, while the remaining 85% of the PD population are classified as sporadic PD (Fig. 2a). However, the distinction between genetic predispositions in familial and sporadic PD is blurry. No single-gene mutation in PD has a 100% penetrance. Instead, most likely, multiple genetic risk factors act in synergy to increase the chances of both familial and sporadic PD. Such genetic susceptibilities interplay with aging and environmental factors in both familial and sporadic PD.

Mounting evidence demonstrates the role of genetic predispositions in PD pathogenesis, and the prevalence and penetrance of each variant start to emerge. Typically, it appears that the most common PD gene variants found in the global population have the lowest penetrance. On the other hand, most penetrant mutations are rare, and most often associated with familial PD. Variants on at least 16 PARK genes, referred to as “causal” because they instigate a relatively high familial inheritance of PD or early-onset PD symptoms, have been identified and are highlighted in Table 1^5,119,132,135,147,168–178^. To estimate the frequency of these mutations in the Parkinson’s population, we examined the published reports from 50 epidemiological studies. The high penetrance and hereditary nature of causal mutations can amplify their occurrence in specific ethnic groups (i.e., Japanese^134^, Chinese^133^, and Ashkenazi Jews^11^). However, globally our analysis shows that the presence of any known causal PD mutation is rare, occurring in less than 2% of the PD population (Fig. 2b). The two most common mutated genes associated with familial PD are *LRRK2* and *PRKN* and are reported in 0.7% and 0.3% of all the people showing PD symptoms, respectively (Fig. 2b). However, the frequency of at least some risk variants (OR > 1.5), which we measured in a large genomic data set including 25,243 total PD cases (combination of NHGRI-EBI catalog and 24 independent genetic studies), appears higher than in the epidemiological studies, which would tend to focus on the variants with the highest penetrance. For example, *LRRK2 G2385R* is present in >10% of the PD patients and *PRKN* exon rearrangements are present in >3% of the patients (Fig. 2c). Our analysis also highlights that known mutations in familial PARK genes confer a broad range of risks to develop PD symptoms. *PRKN(exon rearrangements)*, *LRRK2(G2019S)*, *GBA (L444P, N370S)*, and *CHCHD2(P2L)* appear to be the most penetrant mutations, increasing the chances of getting PD symptoms by up to a factor 14, 10, 8 and 5, respectively (Fig. 2c). However, these penetrant variants are relatively rare, occurring in ~4%, 2%, 5%, and 2% of the PD population, respectively (Fig. 2c). Overall, the data tends to confirm that the most prevalent variants are less penetrant (See DJ-1 variants for example in Fig. 2c). Recent GWAS allowed for the detection of common low penetrance mutations associated with PD. To date, over 44 novel risk loci have been identified and associated with sporadic PD^9,14,141,179,180^. Mutations within these risk loci can be disease modulating (OR > 1) or protective (OR < 1) for PD (Fig. 2d). Despite GWAS and genetic studies pointing to several novel molecular targets and pathways in sporadic PD, many of them remain unconfirmed. The presence of mutations on a single risk locus often poses a low risk for disease, yet mutations in multiple risk loci can collectively be disease modulating. The lack of a preponderant single definitive causative gene for most PD patients is a challenge for both the design of experimental models and the discovery of well-targeted treatments. Novel approaches for disease modeling in vitro are required to unravel the polygenic and complex mechanisms underlying sporadic disease successfully. Brain tissue engineering from patient-derived stem cells provides a unique opportunity to fulfill such need.

**Table 1:** Summary of Parkinson’s genes first identified in familial and early-onset cases of PD and risk factors.

Gene (Chromosomal position)	Gene product	Function	Inheritance	Mutations studied in iPSC studies	Reference (iPSC studies)
SNCA (4q21)	a-synuclein	Suppression of apoptosis, chaperone activity, antioxidation, neuronal differentiation, regulation of DA biosynthesis	AD	A53T; E46K; duplication; triplication	28,30,33–35,41,42,49,55,60,66,73,80,83,84,89,90,
PRKN(6q25.2-q27)	Parkin	Mitochondrial quality control	AR	V324A; T240R; R275W; R42P; −1 bp del 255A; Exon 3–4 del; Exon 2 del; Exon 3/5 del; Exon 6/7 del; Exon 2/4 del; Exon 5/6 del; Exon5 del; Exon 3 del	24,29,31,39,40,42,54,64,68,71,74,76,77,84,88,91,
PINK1 (1p35-p36)	PTEN-induced kinase 1	Mitochondrial quality control	AR	G309D; V170G; Q456X	31,32,52,62,70,71,85,
DJ-1 (1p36)	DJ-1	Protection against oxidative stress	AR	E46D	27
LRRK2 (12q12)	Leucine rich repeat kinase 2	Membrane trafficking, mitochondrial membrane maintenance	AD	G2019S; R1441C; R1441G; I2020T	26,32,37,38,42–44,46–48,50,51,53,56,57,61,65,78,79,82,84,86,87,
ATP13A2 (1p36)	Cation-transporting ATPase 13A2	Cation homeostasis, lysosomal function	AR	N/A	N/A
VPS35 (16q11.2)	Vacuolar protein sorting 35	Recycling of membrane proteins between endosomes and the trans-Golgi network	AD	N/A	N/A
DNAJC6 (1p31.3)	DNAJ subfamily C member 6	Clathrin mediated endocytosis	AR	N/A	N/A
FBXO7 (22q12–q13)	F-box protein 7	Phosphorylation-dependent ubiquitination	AR	N/A	N/A
SYNJ1 (21q22.11)	Synaptojanin-1	Regulation of synaptic vesicle endocytosis	AR	N/A	N/A
PLA2G6 (22q12–q13)	Phospholipase A2, group 6	Phospholipid remodeling, mitochondrial function	AR	N/A	N/A
CHCHD2 (7p11.2)	Coiled-coil-helix-coiled-coil-helix domain 2	Regulation of mitochondrial metabolism under oxygen stress	AD	N/A	N/A
GIGYF2 (2q36–q37)	GRB10 interacting GYF protein 2	Negative regulation of cell growth	AD	N/A	N/A
Omi/HTRA2 (2p13)	High-temperature requirement A2	Neuroprotection	AR	N/A	N/A
VPS13C (15q22.2)	Vacuolar protein sorting 13C	Maintenance of mitochondrial function	AR	N/A	N/A
EIF4G1 (3q27.1)	Eukaryotic translation initiation factor 4 gamma 1	Regulation of mRNAs translation	AD	N/A	N/A
UCHL1 (4p13)	Ubiquitin C-terminal hydrolase L1	Ubiquitin–proteasome system and neuronal survival	AD	N/A	N/A
GBA (1q21)	Glucosylceramidase Beta	Lysosomal function	RF	N370S; L444P; RecNil	30,36,58,59,69,84,
MAPT (17q21.31)	Microtubule-associated protein Tau	Modulates the stability of axonal microtubules	RF	Haplotype H1/H2	25

N/A indicates mutations not studied in iPSC-PD studies.

*AD* autosomal dominant; *AR* autosomal recessive; *RF* risk factor; *del* deletion.

## Studies of PD with patient-derived iPSCs

**Fig. 3:**
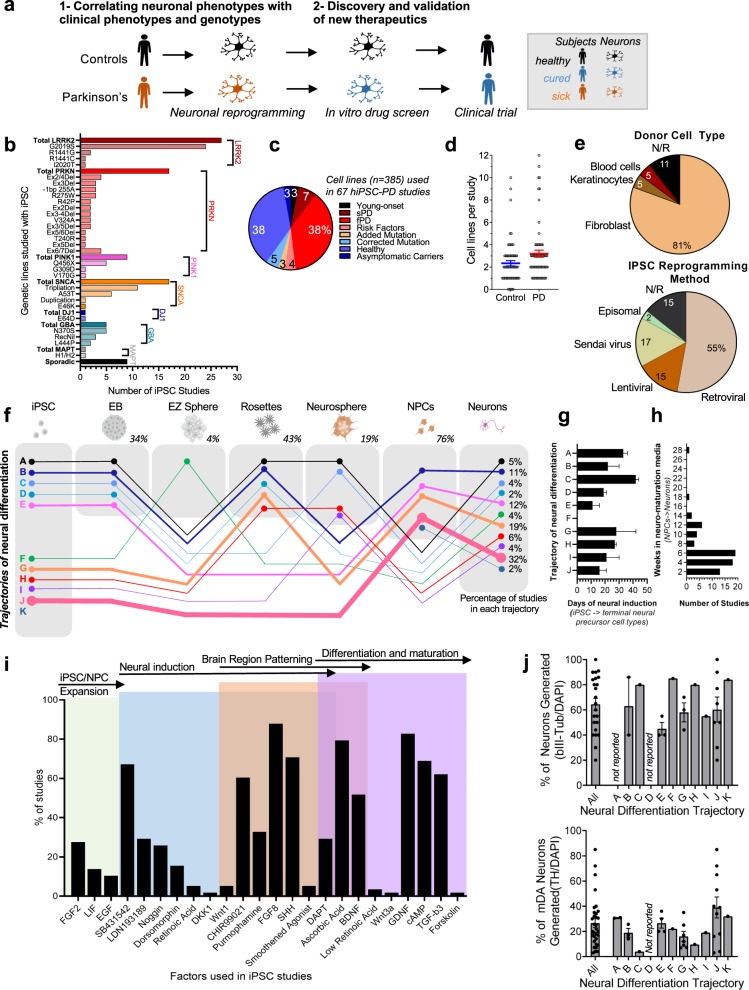
Using brain cells generated from patient-derived iPSC to study PD in vitro. Data from this figure was extracted and analyzed from 67 iPSC-PD studies, refer to “Methods”. **a** A schematic pipeline of in vitro disease modeling and preclinical drug screening with patient-derived brain cells. **b** The number of iPSC studies that used human neuronal lines with corresponding mutations on specific genes associated with PD (also refer to Table 1). Categories in bold and darker bars represent the total number of studies examining that gene. **c** The types of control and PD cell lines are displayed as the percentage of total cell lines. **d** The number of PD and control cell lines used in iPSC-PD studies. Data presented as the mean ± SEM. **e** Donor cell types and reprogramming methods used in hiPSC-PD studies. N/R indicates that details were not reported in these studies. **f** The diagram summarizes the different type of tissue culture trajectories used to differentiate cultures of iPSCs into midbrain neurons. Line thickness and percentages (in the “neurons” box) represent the proportion of studies in corresponding trajectories. The percentage displayed for each intermediate stage shows the proportion of studies that uses the corresponding cell type. EB embryoid bodies, NPCs neural progenitors **g** Neural induction duration indicates the number of days (average + range) required for the generation of terminal neural precursor cell types (last stage before neuronal maturation: NPCs, neurospheres, rosettes, or EZ spheres depending the stages that were skipped) from iPSC. **h** Neural maturation duration indicates the average number of weeks from terminal neural precursor cell type (NPCs or previous stage if NPC stage was skipped) to the neuronal cells used for phenotypic evaluation. **i** Small molecules and growth factors were used at various stages of midbrain dopaminergic neuronal differentiation. Data presented as the percentage of hiPSC-PD studies that report the corresponding factors in the tissue culture media composition. **j** The proportions (mean + SEM) of neurons (bIII-Tub/DAPI) and midbrain dopamine neurons (TH/DAPI) in cultures vary between differentiation protocols and trajectories. Each data point is the average percentage reported in a single study (*n* = 33 independent studies, refer to “Methods”). The first column labeled as “all” groups all the studies regardless of their differentiation trajectories. Relevant immunohistochemistry quantification was not reported in studies using neural differentiation trajectories A and D.

The discovery of iPSC technology^181–183^ has offered the capacity to generate live brain tissue from healthy subjects and patients for studying neurodegenerative diseases^184^. Directed reprogramming and neuronal differentiation of iPSCs allows the study of specific neuronal subtypes. Human-derived neurons offer a unique opportunity for modeling real cases of human genetic diseases in vitro. The ability to generate neurons both from PD patients and healthy control individuals allows the identification of early disease-linked phenotypes and provides a new paradigm for preclinical drug development and validation (Fig. 3a).

Since 2011, over 385 hiPSC lines have been generated from PD patients (*n* = 215) and controls (*n* = 170). Despite the large epidemiological preponderance of sporadic cases of PD (85% sporadic vs. 15% familial PD, Fig. 2a), only a few studies have modeled sporadic PD with iPSCs (Fig. 3b). Majority of iPSC-PD studies modeled LRRK2-G2019S, PRKN exon deletions, PINK1 Q456X, SNCA triplication, and GBA N370S (Fig. 3b). When combined all together, less than 20% of the iPSC disease lines were sporadic and 80% of the disease cell lines modeled single causal mutations occurring in familial PD (Fig. 3c). Extended application of iPSC technology beyond monogenic disease to multigenic sporadic disease is required to reveal cellular features relevant to the majority of people affected by PD. An increasing number of iPSC-based models of neurological and psychiatric disorders show that even complex brain disorders with limited heritability lead to neuronal phenotypes in vitro^185–187^. Despite this current bias in the literature towards familial PD, these pioneer studies are extremely valuable and, in the sections below, we will compare their methods and summarize the overlap and discrepancies of the phenotypes reported in PD neuronal lines.

## How many patient neuronal lines should we use for iPSC models of PD?

This is a crucial question for which a consensus remains difficult to reach. Due to the cumbersome and costly nature of iPSC studies, a balanced trade-off between a large number of neuronal lines and the depth of the analysis is inevitable. For comparison, other kinds of PD patient-control studies vary quite substantially from GWAS generally including thousands of case–control subjects to postmortem brain tissue studies including cohorts of 11 ± 5 patients and 8 ± 6 controls, on average^188^. We report here that current iPSC-PD studies, on average, use five individuals per study (two control and three patient lines) and a maximum of 12 controls and 11 PD line. Fewer than 12% (8/67) of published studies used more than ten cell lines (Fig. 3d)^37,42,56,70,79,84,92^. Interestingly, it has been estimated that sample sizes of 10–30 individuals per hiPSC study may be required to achieve a statistical power of 80%, assuming that the cellular readouts variance is high and the disease effect is small (>0.7 relative heterogeneity, which is the ratio between within-group standard deviation and mean group difference)^189^. In this situation, most iPSC studies of PD may fall below the suggested sample size requirements. However, it is important to note that due to the novelty of iPSC models, predicting the statistical power of a study remains approximative, and the number of lines required highly depends on the variance of the cellular phenotypes obtained with a specific analytical readout. It may be possible to reduce the variance without increasing the number of cell lines. For example, the patients from which the biopsies are taken may be selected based on gender, age, ethnicity, social context (i.e., farmers exposed to pesticides or professional athletes exposed to repetitive trauma), genotypes, and clinical severity of the symptoms. Ultimately, the clinical and genotypical homogeneity of the subjects may reduce the variance of the cellular phenotypes and may justify using a smaller number of cell lines. This may explain the current bias in the literature towards iPSC models of familial cases of PD (Fig. 3b, c). Contrastingly, there is an assumption that high genetic heterogeneity in sporadic disease contributes to a range of cellular phenotypes and therefore requires a higher number of cell lines to obtain statistical power^19,45,190^. However, this assumption might be wrong if multigenic sporadic PD predispositions converge to similar clinical symptoms caused by common brain cell phenotypes. Despite the current limitation of a relatively low number of cell lines used in each study, and regardless whether familial or sporadic patients were included, significant PD phenotypes have been reported, thus demonstrating the value of this research model. However, to translate these results into clinical trials and maximize their chance of success, validation in a larger number of cell lines is desired. Studies including a high number of cell lines will benefit from recent advances in high-content technologies. However, unfortunately, performing in-depth analysis of 10–30 cell lines requires substantial resources that most laboratories do not have access to. The strategy taken in this review to combine the results from several independent studies may be a necessary compromise between in-depth analysis and a high number of cell lines. The optimization and harmonization of tissue culture methods may also be key to facilitate the identification of robust phenotypes across studies and will be discussed below. In addition, new methods decreasing the variance of neuronal phenotypes in iPSC models and decreasing the cost of the analyses are necessary and will also be discussed in the next sections.

## Choosing the right control: healthy matched subjects or genetically edited isogenic lines?

Disease-linked cellular phenotypes are identified by comparing neuronal lines from patients and controls. To date, healthy subjects are the most commonly used control cell lines (Fig. 3c). However, differences in genetic background may give rise to variance in neuronal phenotypes that are unrelated to the disease. Some studies try to address this by using asymptomatic carriers, which are typically siblings or first-degree relatives of the patient^27,28,33,35,57,62,69,70^. Despite reducing genetic variability by around 50%, the limited availability of these controls hampers their use. In addition, asymptomatic carriers may express mild disease phenotypes^57,69,70^, which can increase the threshold for detecting the early disease phenotypes of progressive neurodegenerative disorders in symptomatic carriers. In order to reduce further genetic heterogeneity between control and disease lines, the development of genetic editing techniques such as TALEN, ZFN, and CRISPR/Cas9 has enabled the generation of isogenic lines^191–194^. Differing in only one single known mutant gene, the comparison of isogenic neurons allows the precise analysis of the role played by a specific mutation in disease modulation. Gene editing techniques have been particularly useful for studying monogenic forms of PD. Specifically, restorative isogenic lines of common genetic variants including *LRRK2*, *SNCA*, and *GBA* have highlighted complex roles of these mutations in protein aggregation, autophagy, and lysosomal dysfunction observed in PD^49,55,58^. In addition, PD mutations have been introduced into healthy subject or embryonic stem cell lines^34,43,51,53,66^. Having access to isogenic lines minimizes genetic background variability between patient and control lines, thereby reducing the threshold of detection for disease-related cellular phenotypes. However, considering the low penetrance of most familial PD genes, it may also be argued that the “genetic background” of a patient contributes to the cellular phenotype in a multigenic synergic way.

## Generating relevant neuronal cell types for PD

The cellular reprogramming toolbox for researchers is rapidly expanding and includes a panoply of neuronal differentiation protocols to generate cells representing various brain regions^21^. PD is a debilitating motor system disorder resulting from the selective degeneration of midbrain dopamine (mDA) neurons located in the substantia nigra pars compacta. Protocols have been established to specifically generate dopaminergic neurons and brain cells with a midbrain molecular profile^195,196^.

More than 86% of published hiPSC-PD studies started with donor skin cells (fibroblasts and keratinocytes) and only 5% used blood cells (peripheral blood mononuclear cells, CD34+ cord blood cells or lymphoblastoid cells) (Fig. 3e). More than 70% of iPSCs were generated using integrating viral vectors (retroviral and lentiviral), 17% used non-integrating viral vectors (Sendai virus) and only 2% used viral-free approaches (episomal) (Fig. 3e). Several neuronal differentiation methods used across the 67 iPSC-PD studies were also analyzed in this review^26,36,39,44,86,197–204^. Different methods followed distinct neuronal differentiation trajectories (from iPSC to neurons), involving the generation of various intermediate cell types (Fig. 3f). The intermediate stages may include embryoid bodies (EB, stem cells in suspension), EZ sphere (aggregates of early neural stem cells in suspension), neural rosettes (early neural stem cells with radial arrangements), neurosphere (neural progenitors in suspension), and neural progenitor cells (NPC, neural precursors with bipolar morphology). The generation of cell types such as EBs, neural rosettes, and NPCs mimics various stages of in utero neurogenesis^21^. Few protocols also generated unconventional cell types such as EZ spheres^202^ or neurospheres^205^. Such alternatives aim to eliminate laborious differentiation steps involving EB formation and manual picking of rosettes. On average, the generation of precursor cell types such as EZ spheres, neurospheres, or NPCs can range from 11 to 45 days (Fig. 3g) and neural maturation can range between 2 and 28 weeks (Fig. 3h). Temporal manipulation of small molecules and growth factors is used to induce neural induction, selective brain region patterning, differentiation, and maturation (Fig. 3i). Diverse combinations of molecules and choice of neural reprogramming trajectory can increase the efficiency and specificity of mDA neuronal differentiation. Midbrain DA neurons can also be generated using only small molecules or with the aid of lentiviral vectors^70,86,200^. Varying differentiation protocols may influence the quality of cells. For example, neurons generated from floor-plate intermediates exhibited PD phenotypes, while cells generated on a stromal feeder layer from neural rosette intermediate did not^31^. Therefore, in some cases, phenotypic variability in iPSC-PD studies can be a consequence of varying neural differentiation protocols.

## Harmonizing rigorous protocols across studies

Regardless of the differentiation method, cell cultures can be widely heterogeneous. On average, iPSC-PD studies, which use midbrain patterning protocols, yield 64% neurons and 27% dopaminergic neurons of the total cells (Fig. 3j). Proportions of neurons (bIII-Tub+) and dopaminergic neurons (TH+) vary vastly between studies even when using identical differentiation techniques (Fig. 3j). Quantitative analyses conducted at discordant timepoints along the maturation process may further contribute to differences in reported proportions of cell type between studies. In addition, arbitrary thresholds of immunocytochemical staining may alter the quantification of cell type numbers reported. Some studies exclusively focus on the analysis of dopaminergic neurons, but many others consistently report the presence of astrocytes, neural stem cells and multiple midbrain neuronal subtypes (i.e., glutaminergic and GABAergic) in their cultures^41,74^. Mixed cultures of human neurons and glia are more accurate in representing the neurophysiology of the human brain in vivo but add some variance in the model, which needs to be taken into consideration during experimental planning. For disease modeling, inconsistencies in both study design and analyses between laboratories can hinder the reproducibility of the results. The artificial nature of reprogramming protocols makes it difficult to agree on the superiority of a single protocol. As the field progresses, the gap between the quality of in vitro and in vivo brain tissue will tighten. The methods generating brain tissue as close as possible to their in vivo counterpart will become gold-standard methodologies, which will help to harmonize culture models between laboratories and may help expedite the clinical translation of the most robust and reproducible results. However, in some cases, for example large-scale studies requiring fast and cheap protocols, a trade-off between brain tissue quality and ease of the methods may be necessary.

An alternative and complementary strategy is to use a top-to-bottom approach, which consists of identifying homogenous cell types of interest in mixed cultures before comparative analysis^206^. The identification of desired cell types can be achieved with molecular or functional analyses. The selection or enrichment of specific cells, for example electrophysiologically mature dopaminergic neurons, can be achieved during the cellular reprograming process or right before the final comparative analysis. Fluorescence-activated cell sorting (FACS) can facilitate the removal of non-neural cell types to focus the analysis on a pure population of mDA neurons^68,69^. However, homogeneity based on single markers may be insufficient. Recent large-scale single-cell transcriptomics revealed the complexity of multiple cell types in neuronal culture as well as brain in vivo^207^. We and others also reported that batch or tissue culture protocol variations, such as passage number or basal media, can generate a mixture of neurons at various electrophysiological states of maturity^206,208^. To ensure an unbiased comparison restricted to functionally mature neurons, a thorough analysis of neuronal properties using electrophysiological techniques is necessary. The recent development of Patch-Seq technology, which combines single-cell RNA-sequencing (RNA-seq) and patch clamping, enables electrophysiological, transcriptomic, and morphological profiling of single neurons^206,209,210^. The multimodal characterization of cellular subtypes can be used to eliminate functional immaturity bias in iPSC-based disease modeling^206,210^.

## How environmental factors and aging can be recapitulated in vitro

An obvious limitation of in vitro models is the lack of environmental context. The influence of nongenetic factors is not recapitulated in the basal phenotype of patient-derived neurons. For example, the influence of head trauma of a boxer with sporadic PD will not be recapitulated by default in reprogrammed neurons. An alternative would be to transplant the patient-derived neurons in animals and simulate the trauma on the animal. Similarly, influence of decades of aging of the human brain is difficult to reproduce in vitro in a few months within the boundaries of feasible experimental design. Brains in a dish will always be an imperfect experimental model. However, many tricks can be used to recapitulate the environmental and aging stress in vitro. Table 2 summarizes a list of reagents that have already been used in iPSC neuronal culture to mimic oxidative stress, proteostatic stress, mitochondrial stress, synaptic stress, ER stress, inflammation, and cellular aging. An interesting example is progerin, a truncated form of lamin A associated with premature aging. Increasing the expression of progerin in iPSC neurons can recapitulate at least some aspect of cellular aging in vitro^71^. Human iPSC-derived dopamine neurons overexpressing progerin displayed specific phenotypes such as neuromelanin accumulation. In addition, PD patient-derived neurons revealed disease-related phenotypes that required both genetic susceptibility and induced-aging in vitro^71^.

**Table 2:** Cellular stressors used in hiPSC-PD models.

Type of stressor	Reagent	What is it?	Mechanism of action	Environmental and biological relevance
Proteostatic	MG132	Peptide-aldehyde (proteasome inhibitor)	Initiates apoptosis and reduces degradation of ubiquitin-conjugated proteins by inhibiting 20S proteasome activity	*
	Cycloheximide	Naturally occurring fungicide	Inhibits global transcriptional function	*
	Lactacystin	Proteasomal inhibitors	Inhibits ubiquitin–proteasome proteolysis pathway	*
	Epoxomicin	Naturally occurring proteasome inhibitor	Inhibits protein degradation by inhibiting chymotrypsin-like proteasome	Used to induce PD-like symptoms in animal models
	Leupeptin	Naturally occurring protease inhibitor	Inhibits lysosomal proteolysis by inhibiting cysteine protease	Used to induce aging associated degeneration of neural processes in animal models
	Ammonium Chloride	Weak Base	Inhibits lysosomal proteolysis by altering pH of lysosomes	Component of fertilizers
	Bafilomycin	Toxic macrolide antibiotic	Inhibits autophagy by preventing maturation of autophagic vacuoles. Targets vacuolar type H+-ATPase causes lysosome neutralization and inhibition of autophagosome and lysosome fusion.	*
	Chloroquine	Prototype anti-malarial drug	Inhibits autophagy by increasing lysosomal pH and preventing fusion of autophagosomes and lysosomes.	*
	Rapamycin	Macrolide compound	Increases autophagy by inducing autophagosome formation via inhibition of mTOR	*
Mitochondrial	FCCP/CCCP	Protonophore (respiratory chain uncoupler)	Reduce cell respiratory capacity by inhibiting mitochondrial cell volume and membrane potential	*
	Rotenone	Insecticide	Inhibits mitochondria respiration and ATP generation via inhibition of mitochondrial complex I	Insecticide associated with PD epidemiology
	Valinomycin	Antibiotic/Ionophore	Induced loss of mitochondrial membrane potential and mitochondrial translocation via facilitating potassium transfer across lipid layers	*
	Oligomycin	Antibiotic	Blocks ATP synthesis via inhibition of ATP synthase preventing oxidative phosphorylation of ADP	*
	Antimycin A	Antibiotic	Inhibits mitochondrial electron transport by inhibition of complex III	*
	Paraquat	Herbicide	Increases ROS	Herbicide associated with PD epidemiology
	MPTP	Impure by-product of recreational drug design	Induces cell death due to ATP depletion via inhibition of mitochondrial complex I	Causes Parkinsonism in users
	Maneb	Fungicide	Alters cellular redox reactions by catalysing DA oxidation and chelation of metals	Fungicide used to generate PD in animal models
	Concanamycin A	Antibiotic	Inhibits mitophagy by preventing acidification of organelles via inhibition of H+-ATPase	*
Synaptic	KCl	Metal halide salt	Induces dopamine release by increasing calcium response	KCl found in some foods and used as a salt substitute
	Glutamate	Neurotransmitter	Activates extra-synaptic and synaptic excitatory glutamatergic receptors via AMPA, NMDA Kainite receptors, and alterations in intracellular calcium	Main component of food flavourer monosodium glutamate (MSG)
	Caffeine	CNS stimulant	RyR agonist that increases the calcium release from the calcium-sensitive internal stores	Widely consumed psychoactive drug present in many foods
	Colchicine	Microtubule depolarizing agent	Inhibits microtubule polymerization by binding to tubulin	Promote neurite retraction in vitro
Oxidative	Hydrogen Peroxide	Strong oxidizer	Directly increase oxidation and ROS	Induce oxidative stress linked to aging
	No antioxidants	Removing antioxidants from neuronal culture media	Reduce the prevention of the oxidation of molecules	Antioxidants in food
	6-OHDA	Synthetic compound (enters neurons via neurotransmitter uptake transporters)	Produce ROS specifically in dopaminergic and noradrenergic neurons; inhibition of mitochondrial complexes I and IV	Selective destruction of DAn used to generate PD in animal models
	Dopamine	Neurotransmitter	Induced dopamine toxicity by increased dopamine oxidation by monoamine oxidases (MAO) in cytoplasm	Mimic chronic use of Levodopa
	Cadmium	Transition metals	Induce oxidative stress by depleting cells’ antioxidants	Heavy metals present in contaminated air, water. soil and food
	Exogenous a-synuclein	Major component of Lewy bodies	Increase basal ROS levels	Mimic effect of a-synuclein accumulation in vitro
ER	A23187	Calcium ionophore	Induced ER stress by increasing cytosolic calcium concentrations	*
	Brefeldin A	Fungal metabolite	Inhibits intracellular protein transport by preventing association of COP-I coat to the Golgi membrane	*
	DTT	Reducing agent	Induces ER stress by preventing inhibition of disulfide bonds	*
Age-inducing	Progerin	Truncated version of the lamin A protein	Increases the frequency of unrepaired double-strand breaks in DNA	Mimics aging processes
Inflammation	IL-17	Hematopoietic growth factor (cytokine)	Induces inflammation through IL-17–IL-17R signaling and activation of NFkB	Naturally occurring inflammatory cytokine

These compounds were used to stress cells to explore mechanisms of PD not to cause PD in models.

An asterisk indicates a synthetic compound not naturally occurring or that does not have environmental or biological relevance.

## Human iPSC studies of PD highlight converging molecular and cellular pathways across genetic subgroups

**Fig. 4:**
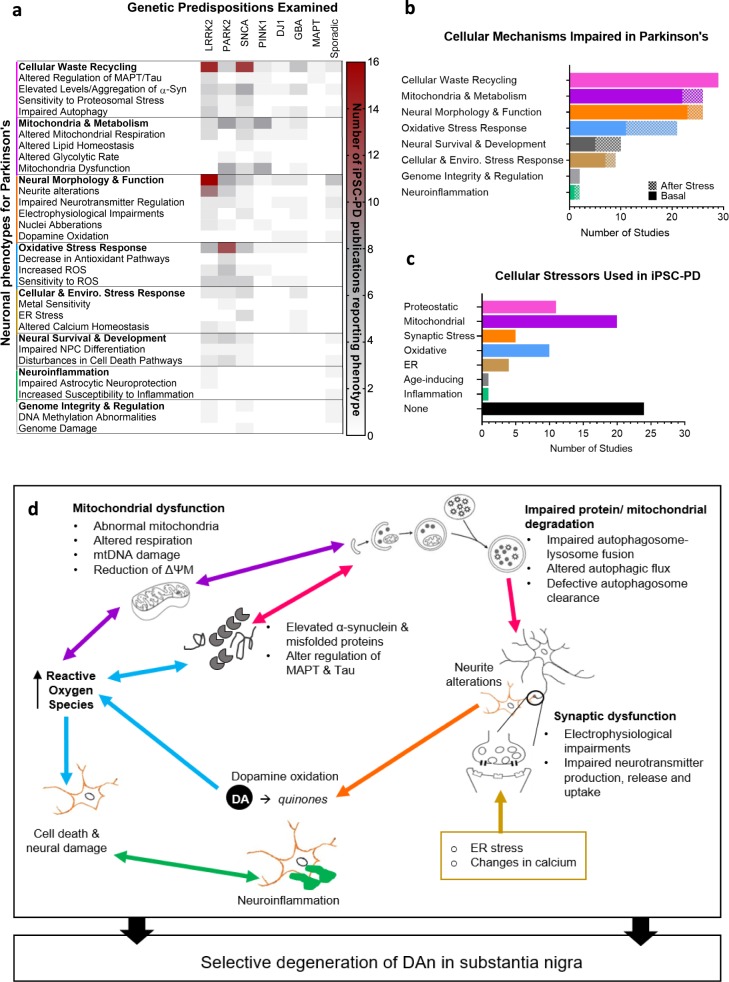
Phenotypic insights from iPSC studies of Parkinson’s disease. **a** A heatmap representation of neuronal phenotypes reported in genetically heterogeneous PD lines examined in 67 hiPSC studies. Categories in bold represent a sum of all sub-phenotypes. Reported absences of phenotypes are not represented in the figure. **b** Summary of the impairments in cellular mechanisms which were reported in iPSC-PD studies. Data represented as total number of studies reporting impairment with or without induced stress (“after stress” or “basal”, respectively). **c** Types of artificial cellular stressors used across hiPSC-PD studies (refer to Table 2). Data presented as the number of studies that used stressor to induce or investigate PD. **d** Schematic representation of crosstalk between cellular mechanisms involved in PD pathogenesis.

Our analysis of 385 iPSC-derived cell lines from 67 published studies reveals that many PD neuronal phenotypes are shared between genetically heterogeneous familial and sporadic patients (Fig. 4a). Notably, impairments in mechanisms involved in cellular waste recycling, mitochondrial function, neuronal morphology and physiology, and sensitivity to reactive oxygen species (ROS) are most common across patient lines with varying genetic predispositions (Fig. 4b). The studies measured cellular phenotypes that occurred either spontaneously or in response to chemicals mimicking cellular aging and stress (Table 2 and Fig. 4a, c). It is important to note that the frequency of reported phenotypes in our meta-analysis may be biased because only few studies (~19%, 13/67) reported negative results (absence of phenotypes)^31,32,36,37,40,45,48,52,59,64,74,76,86^. In addition, most cell lines were not systematically phenotyped without prior hypothesis and thus, there is likely to be an ascertain bias in these phenotypes. Less hypothesis-driven multimodal or omics analysis will help to address such bias^41,72,76–80,87,90^. Phenotypes caused by genomic predispositions allude to crosstalk and impairments in multiple pathways that act collectively to mediate selective degeneration of dopaminergic neurons in the substantia nigra (Fig. 4d) and will be discussed in detail below.

### Genetic predispositions reduce mitochondrial respiration in patient-derived neurons

Epidemiological studies outlined in Fig. 2 have pointed to several genes involved in mitochondrial function and recycling (e.g., *CHCHD2, PRKN, PLA2G6, VSP13C, PINK1, LRRK2*; see also Table 1). An increase in dysfunctional mitochondria was identified in 26 independent hiPSC-PD studies using *LRRK2*, *PRKN*, *PINK1*, *GBA*, and *SNCA* patient-derived neuronal lines^24,27,31,32,37–39,52,55,57,59,61,62,64,67,68,70,71,73,74,77,85,89,90^. Neurons derived from PD patients displayed an increase in mitochondrial copy number^62^ and a greater proportion of abnormal mitochondria^31,39^. Abnormal mitochondria exhibited either signs of enlargement^31,71^, shortening^32^, thinning^64^, irregular cristae structures and network^59,74^, or fragmentation^24,70^. Mitochondria in the proximal axon of neurons from individuals carrying the *LRRK2 R1441C* mutation were 20% shorter than individuals with *LRRK2-G2019S* mutations^32^. An increase in bidirectional mobility of mitochondria was also reported in *LRRK2* lines but not in *PINK1*^32^. Overall, cellular respiration and metabolism were altered in neuronal lines derived from *LRRK2*, *PRKN*, *SNCA, PINK1*, *DJ-1, and GBA* patients^27,37,55,58,59,61,67,70,73,83,85^. Basal mitochondrial respiration is consistently compromised across PD lines, exhibited as reductions in maximal oxygen consumption rate, diminished ADP and ATP levels and NAD+/NADH redox states^27,55,58,59,61,67,73,85^. Impaired mitochondrial respiration was also associated with an increase in mitochondrial ROS and lysosomal hyperactivity^67^. The glycolytic rate of patient-derived neurons was higher in *PINK1*^70^ and *PRKN* lines^77^. This was not observed in the transcriptomic analysis of *SNCA* lines^90^. An increase of passive proton leakage from the inner membrane was reported in *PINK1* mutant neurons but not in *LRRK2*^32^. In addition, the degradation and translocation of mitochondrial membrane proteins such as PARKIN and MIRO1 were impaired in *LRRK2* and *PRKN* neurons^38,62,73^.

Overall neuronal metabolism may be impaired by endogenous dysfunction of mitochondria^25,31,37–39,52,55,57,59,61,64,67,68,70,73,74,77,85,89,90^, or indirect external stressors^24,32,62,71,90^, or by abnormal recycling of damaged mitochondria^38,39,52,68^. For example, some patient neurons displayed signs of impaired mitochondrial quality control related to aberrant mitophagy^38,52,68^, mitochondrial DNA damage^37,57^ and consistent decreases in mitochondrial membrane potential^24,38,67,83,85^.

Genetic predispositions that reduce the constant supply of energy to neurons ultimately disrupt the functioning of brain circuits. Mitochondrial impairments can confer increased neuronal vulnerability to oxidative, nitro-oxidative stress, and metal ions^24,32,39,55,58,67,68,74^. The high metabolic demand of substantia nigra neurons might explain their selective degeneration^211^.

### Impaired oxidative stress-buffering capacity increases neuronal susceptibility to cell death in PD

Impairments in multiple cellular mechanisms increase oxidative burden in patient-derived neurons with mutations in *LRRK2*, *PRKN*, *PINK1*, *GBA*, *DJ-1*, and *SNCA* (20 studies)^24,27–29,32,33,38,40,42,46,48,53,55,58,68,77,88,89^. Elevated basal ROS levels reported in patient-derived neurons increase cell susceptibility to cell death such that patient lines treated with oxidative and mitochondrial stressors (i.e., H_2_O_2_, rotenone and paraquat) exhibit significantly higher levels of the cell death marker caspase-3 compared with healthy controls^27–29,33,39,46,68,77,88^. Notably, antioxidant buffering in patient lines is reduced, marked by a low level of antioxidant enzymes and the downregulation of antioxidant pathways such as NRF2 and target gene *NQO1*^29,39,77^. In addition, ten studies, which did not report oxidative stress phenotypes in basal conditions, reported and increase vulnerability when oxidative stress was induced with chemicals^24,31,32,40,42,48,53,55,58,89^. This suggests that PD genetic predispositions could be compensated by upregulating endogenous antioxidant pathways or providing antioxidant supplements. Several clinical trials testing antioxidants treatments are ongoing. However, unfortunately, so far, clinical trials failed to show that antioxidants such as MitoQ significantly slow down the progression of symptoms^212^. Antioxidants may need to be combined with other treatments for more positive outcomes.

### Genetic predispositions increase the probability of protein aggregation in PD neurons

Alpha-synuclein and Tau protein accumulation constitute the most common phenotype reported in 28 studies investigating *LRRK2*, *PRKN*, *PINK1*, *GBA*, *SNCA*, and *MAPT* lines^25–31,34–36,39,41,43,46,48–51,53,55,56,58,60,65,67,69,73,83^. The upregulation *MAPT* (microtubule-associated protein tau) transcripts were associated with protein aggregation, which impaired axonal mitochondria movement and induced neurite aberrations in patient neurons^25,53,213^. The accumulation of α-synuclein and Tau has also been noted in the varicosities of contorted and fragmented axons of patients^41^. Elevated expression of *SNCA* mRNA was associated with the dysregulation of oxidative stress, protein aggregation, and cell death regulatory genes, proposed to induce or increase selective sensitivity of mDA neurons to these factors^28,29^. Alpha-synuclein protein levels were also elevated in patient-derived neurons^27–30,34–36,39,41,46,50,51,55,56,65,69,73,83^. Notably, the level of soluble^27^ and monomeric^28,46^ conformations of α-synuclein were increased. Increased events of α-synuclein oligomerization^73^, elevated levels of insoluble oligomers^27,31,55^ and the formation of inclusion bodies^65^ were also reported. Neurons derived from *SNCA* mutant iPSCs^55^ displayed an increase in the phosphorylation of α-synuclein at residue S129; this was not detected in *LRRK2*^51^ derived neurons. Alpha-synuclein oligomers mediated neurotoxicity through metal redox reactions and increased the formation of fibril aggregates, forming Lewy bodies^33^. Nevertheless, neurotoxicity mediated by protein aggregation can be a consequence of impairments in various contributing cellular mechanisms including inflammation, mitochondrial function, autophagy and stress, suggesting a role in late PD pathogenesis^36,39,44^.

### Dysregulation of autophagy and protein ubiquitination contributes to neurodegeneration

Across 15 hiPSC studies, evidence of impaired protein degradation in *LRRK2*, *PRKN*, *GBA*, *DJ-1*, *SNCA*, and sporadic PD lines has been reported^26,27,29,36,45,48–50,53,56,58,63,69,75^. Unbiased pathway analysis of cysteine-modified proteins revealed an enrichment of proteins involved in the ubiquitination and removal of unfolded protein^76^. Accumulation of autophagosomes and increased expression of key autophagy regulators (i.e., beclin1, p62) were also consistently noted in PD neurons, suggestive of an upregulation of autophagy initiation^26,36,48,49,56,74^. Increased protein levels of the autophagy marker LC3B-II in *LRRK2 I2020T* were associated with increased markers of protein oxidation^48^. However, reductions in microtubule-associated protein light chain 3 (LC3) and lysosomal marker colocalization reflective of unsuccessful autolysosome formation and maturation implied disturbances in autophagy progression^45,56^. Increasing genetic, epidemiological, and clinical studies draw biochemical and cellular links between PD and lysosomal storage disorders^214,215^. Mutations in lysosomal genes such as *GBA, SMPD1*, and *ATP13A2* have been shown to impact the bidirectional feedback loop for processing and clearance of α-synuclein in PD^124,146,216^. Signs of enlarged lysosomal compartments paired with decreased lysosomal enzymatic activity suggested that poor autophagy completion contributed to protein aggregation in PD^27,36,47,58,69,92^. Interestingly, lysosomal impairment in sporadic patient-derived neurons was observed at a later timepoint (180 days) compared with *DJ-1* mutant neurons (70 days)^27^. Young-onset iPSC models of PD revealed cycloheximide treatment specifically slowed the degradation rate of α-synuclein^92^. The dysregulation of ubiquitin genes also increased the susceptibility of patient neurons to proteasome stress^29^. Dysregulated autophagy and ubiquitin–proteasome mechanisms were associated with an increase in extracellular α-synuclein in patient’s dopamine neurons^36,58^. In addition, patient-derived neurons exhibited poor ability to degrade mutant *LRRK2* protein compared with wildtype^50^. LRRK2 (G2019S) mutant protein displayed enhanced-binding to the lysosomal membrane and prevented the assembly of chaperone-mediated autophagy^50^. The inhibitory effect resulted in decreased degradation of long-lived proteins, which may result in unwanted protein–protein interactions mediating neurodegeneration^50^. PD genetic predispositions favoring either aberrant protein degradation or accumulation in patient-derived neurons were associated with increased vulnerability to proteasomal and oxidative stresses, and contributed to accelerated neurodegeneration^27,29,48,53,58^.

### Patient-derived neurons exhibit morphological signs of neurodegeneration

Fifteen hiPSC-PD studies investigating *LRRK2*, *PRKN, SNCA*, and sporadic patient-derived lines reported alterations in neurite process morphology^26,42,45,49,51,53,54,56,60,65,67,71,76,82,88^. Consistently, studies reported reductions in neurite length, branching, and network complexity^42,45,49,51,53,54,56,60,65,67,71,76,82,88^. However, incidences of hyperbranching^26^ and lack of neurite alterations have also been reported^37^. In addition, irregular presynaptic membranous inclusions and dystrophic synaptic structures were reported in a neuronal model of synucleinopathy^73^. The exact mechanism underlying neurite alterations in PD neurons remains unclear and can be attributed either to dysfunction in neurite morphogenesis or increased neurite deterioration^42^. Elevation in autophagic flux, in response to α-synuclein and Tau accumulation, was associated with the reduction of neurite structure and outgrowth in patient lines^49,53^. Induction of the initiation of autophagy or inhibition of its completion also greatly exacerbated the neurite alterations in patient dopamine neurons^26,56^. Microtubule instability induced by the depolymerizing agent colchicine and loss of *PRKN* function also resulted in neural aberrations, though not specifically in TH+ cells^54^. Both autophagy and microtubule polymerization play key roles in neurite injury, growth and axonal formation, and impairments in either of these mechanisms can mediate the neurite alterations observed in PD neurons^217,218^.

### Synaptic dysfunction increases dopamine oxidation in PD leading to neurodegenerative processes

Twelve human iPSC studies examining *LRRK2*, *SNCA*, *GBA*, and sporadic PD reported signs of impaired synaptic function^27,31,40–42,47–49,56,60,69,71,91^. Dysregulation of genes involved in synaptogenesis, synaptic vesicle mechanisms, synaptic transmission, and regulation of neurotransmitter release have been identified in *SNCA*, *LRRK2*, and *PRKN* patient-derived lines^41,42,49,92^. *LRRK2 I2020T* mutation decreased the spontaneous calcium-dependent release of dopamine^48^ but not *PRKN* mutant^40^. The loss of PARKIN in patient midbrain neurons has been shown to downregulate dopamine reuptake machinery^40^. Downregulation of synaptic proteins involved in synaptic vesicle endocytosis was associated with an increase of cytosolic dopamine^47^. Elevated levels of cytosolic dopamine mediate dopamine toxicity and contribute to neural damage and degeneration^27,31,40,69,75^. Excessive oxidation of dopamine by variants of monoamine oxidases in the cytoplasm increases the formation of ROS and toxic quinones, contributing to neurodegeneration^40,69^. Disruption of calcium homeostasis and endoplasmic reticular (ER) stress may mediate excessive neurotransmitter release. RNA-seq analyses also revealed an enrichment of genes associated with ER stress in PD patient-derived neurons^80,90^. More than 50% of generated patient lines overexpressing α-synuclein displayed signs of impaired electrophysiological activity, most notably demonstrated by reduced action potentials upon stimulation^49^. Neurons derived from *SNCA, LRRK2, GBA*, and sporadic patients also exhibited delayed or the absence of firing synchronicity, a decrease in the number of active channels and firing rates, as well as reduced spontaneous activity^41–43,49,56,69^. It has been suggested that, in vivo, neurons may go successively through hypo- and hyperactive phases, possibly as a homeostatic response to the cytotoxic effect of synaptic dysfunction^219^. Such phases remain to be described with human iPSC-derived neurons in vitro, but multiple genetic predispositions appear to directly or indirectly impair synaptic function, which contributes to a vicious neurodegenerative cascade.

### Neuroinflammation exacerbates neurodegeneration in sporadic PD

Midbrain neurons derived from sporadic patients showed increased susceptibility to the effects of adaptive immune cells^72^. Sporadic patient neuronal lines co-cultured with T-lymphocytes exhibited substantial signs of cell death mediated by IL-17–IL-17R signaling and activation of NFkB^72^. Similarly, IL-17 treatment resulted in increased neuronal death^72^. Inflammation in the central nervous system and periphery are key hallmarks of PD^220^. Increasing evidence implicates the role of microglia in neuronal loss, though the underlying mechanisms remain to be determined^221,222^. RNA-seq analysis of astrocytes derived from *LRRK2-G2019S* iPSCs highlighted dysregulation in genes involved in the extracellular matrix, which may reduce the neuroprotective capacity of astrocytes in PD^78^. Investigating the role of neuroinflammation in patient-derived microglia may also contribute to the understanding of the selective vulnerability of mDA neurons in sporadic and late-onset PD^223^.

### Genetic predispositions reducing differentiation yield of mDA neurons

In vitro neural development was impaired in neural lines derived from patients carrying *LRRK2, PRKN, SNCA*, and sporadic mutations^43,49,74,93^. In four independent studies, the differentiation potential of neural progenitor cells derived from patients was significantly reduced, demonstrated by low yields of neurons in comparison with control lines^43,49,74,81,94^. A recent review presented the idea that PD is attributed to significant neurodevelopmental defects, which may increase the susceptibility for disease onset^224^. If confirmed, identifying genetic predispositions that contribute to early developmental defects in iPSC-PD may assist the development of novel PD therapies. However, these phenotypes may appear in conflict with other studies^53,55,76^ capable of generating functional neurons from cell lines with similar mutations. The differences could be due to varying protocols, which may be more or less stressful for the cells.

### Epigenomic alterations linked with PD in patient-derived neurons

The ability to capture unique epigenomic alterations associated with PD remains an important challenge. Reprogramming fibroblasts to iPSCs may erase age-associated^225^ and naive epigenetic signatures which could contribute to sporadic PD pathophysiology^226^. However, an epigenetic phenotype was reported in iPSC-derived PD patient neurons^79,89^. Neuronal lines derived from LRRK2 and sporadic patients exhibited epigenomic alterations when compared with healthy controls^79^. Hypermethylation was prominent in gene regulatory regions associated with the downregulation of transcription factors FOXA1, NR3C1, HNF4A, and FOSL2^79^. Interestingly, *LRRK2* mutant and sporadic PD patient neurons shared similar methylation patterns, which were absent in the original donor fibroblasts^79^. A spontaneous increase in the number of DNA strand breaks and genomic damage^89^ in PD patient-derived neurons could indirectly impact genomic regulation.

## Do iPSC-PD models exhibit similar neural phenotypes to those observed in rodent models?

The use of rodent models to simulate human pathologies associated with PD has been extensively reviewed^227–230^. PD pathogenesis is mostly induced in animal models with 6-OHDA, rotenone, MPTP, paraquat, and amphetamine^231^. These models induce substantia nigra dopaminergic neuron death through inhibition of mitochondrial function and increasing production of ROS^229^. Rats and mice systemically exposed to rotenone and paraquat exhibited signs of Lewy body formation^232,233^. Knockout and transgenic rodent models are designed to investigate the consequential pathological impacts of known PD genetic mutations. Genetic mouse models have provided a platform for the functional studies of proteins associated with PD such as PARKIN, LRRK2, DJ-1, and α-synuclein^234,235^. However, the human genetic background of idiopathic PD cannot be fully recapitulated with animal models.

iPSC-PD patient-derived neurons displayed a reduction in striatal dopamine release, a reduction in neurite complexity, an increased in tau phosphorylation, impairments of dopamine neurotransmission, autophagy, and mitochondrial abnormalities (Fig. 4a, b). These impairments were also observed in transgenic mice PD models^236–238^. Such correlation between models is interesting, even though some of the hypothesis-driven studies in iPSC neurons may have been biased by previous animal studies. IPSC models can also shed light on cellular phenotypes absent in animal models. For example, dopamine oxidation was present in PD iPSC-derived neurons but was not observed in neurons derived from *DJ-1 KO* mice iPSC^27^. Any model has its advantages and limitations and animal and patient-derived neuronal models complement each other. Animal models provide the organism context that is lacking in tissue culture models, whereas patient-derived neuronal models more accurately represent human genetics, which may in turn increase chances of translational success.

## Omics analysis of patient-derived PD neurons

To date 10/67 iPSC-PD studies analyzed have used proteomic, transcriptomic, or epigenomic profiling to phenotype PD patient-derived neurons^41,72,76–80,87,90^. Omics analyses may be less biased and data-driven as opposed to purely hypothesis-driven^239^. Data from omics studies can also help to describe biological relationships between complex intertwined cellular pathways and identify relevant druggable molecular pathways.

Genes associated with clinical PD phenotypes such as abnormal nervous physiology phenotypes, abnormal motor capabilities, coordination, and movement phenotypes were dysregulated in iPSC neurons derived from *LRRK2-G2019S* patients^87^. RNA-seq and proteomics analysis also revealed a heavy dysregulation of genes and proteins associated with mitochondrial function, protein ubiquitination, unfolded protein response, ER/calcium regulation, and oxidative stress in PD patient-derived neurons in comparison with healthy controls^41,69,72,77,78,80,84,87,90^. In addition, the epigenome in PD-derived lines was described as uniquely aberrant compared with healthy controls^79^. Together, omics studies highlighted that the proteomic, transcriptomic, and epigenomic profiles of PD patient-derived neurons exhibited disease-specific alterations, which can also correlate with other neurological diseases^41^. Identifying genes that are consistently dysregulated in the same direction in PD genetic lines across independent studies will help to confirm the key common pathways involved in PD pathogenesis. Much work remains to be done in this field. New human molecular insights into PD pathogenesis will help form a stronger foundation for therapeutic development, and may also benefit other neurological diseases.

## Will findings from PD iPSC models translate to human clinical trials?

One of the most exciting applications of patient-derived iPSC models of PD is to validate pharmacological treatments before clinical trials. The field is still at the stage of improving human brain tissue engineering, and many different protocols are being tested and developed. However, the need for progress in clinical translation for brain disorders is extremely high, and there is no time to wait for brain tissue models to be perfect. Pioneering iPSC studies pave the road to success and identify limitations which help the community to reach a consensus on the minimal requirements to model brain disorders in vitro most accurately. It seems essential to improve the efficiency of reprogramming and differentiation protocols while trying to make those models as physiological and realistic as possible^208,240^. Some concerns are raised that in vitro neuronal development, maturation and function might be too artificial, suggesting that the model may overlook some of the critical processes that occur in vivo. Nevertheless, some defects observed in iPSC-derived neurons have already been confirmed in human postmortem brain tissues^39,43,241–243^. Although this is very encouraging, it is unclear whether significant in vitro phenotypes that cannot be confirmed in postmortem brain tissue should be disregarded. Most postmortem brains also have technical limitations and may represent later stages of the disease, whereas iPSC models may represent earlier stages, preceding neurodegeneration.

Given the apprehensions that in vitro studies may be too artificial, human iPSC-derived neural progenitors may be transplanted into animal brains^244–247^. Besides ethical barriers, xenografts also raise the possibility that the healthy host tissue compensate for the impairment of the transplanted cells. Yet, if the phenotypes observed in vitro are recapitulated in vivo, pharmacological treatments could be assessed in a systemic environment, with much more realistic dosage and administration methods.

## Concluding remarks

PD is increasingly described as a spectrum disorder, with patients experiencing a multitude of motor and non-motor symptoms in a unique way. Similarly, PD genetic predispositions are broad. Hundreds of gene variants increase the risk of PD, but no genetic mutation causes a complete penetrance. We estimated the prevalence of each of the most penetrant mutations (increasing risks by >5 times) to occur in <5% of the global PD population. Despite the broad representation of genetic predispositions in PD patients, our review of current studies using iPSC-derived brain cells demonstrates commonality in cellular impairment susceptibilities. Independent reports highlight a recurring theme around dysfunction of mitochondria, proteasomal mechanisms, synapses, inflammation, and oxidative stress regulation. Constitutive and simultaneous dysregulation of multiple pathways can become overbearing, resulting in accelerated neurodegeneration. As a result, there may be an exaggerated emphasis on pathways which may represent later stages of pathogenesis. Future work will further establish the interdependence of these cellular functions and will help to isolate the initial cause from the downstream consequence of a cellular phenotype. It is also possible that current studies are biased towards studying already known or arbitrarily chosen phenotypes, and the integration of multi-omics analysis will help address this issue. Further optimization of the brain tissue engineering methods will also reduce the threshold for detecting disease-related phenotypes from tissue culture artefacts, facilitating the identification of early cellular phenotypes. Future studies also will have to include the analysis of larger pools of patients including sporadic PD with genetic predispositions more representative of the epidemiology.

The rapid expansion of iPSC disease modeling studies of PD is exciting. Altogether, the current work reviewed here suggests that a neuroprotective therapy, which will stop the neurodegeneration in people living with PD, will most likely require to target multiple pathways at once. The prospect of investigating the impact of multigenic predispositions on brain cell functions will provide information on key modulators of neurodegeneration in PD, and preclinical data for more personalized medicine.

## Methods

### Meta-analysis of epidemiological PD studies

Data from Fig. 2a was obtained from epidemiological studies sourced from PubMed and Google Scholar search engines using keywords “Parkinson’s disease”, “prevalence”, “incidence”, and “epidemiology”. Twelve international studies^94,157–167^ including a total of 5650 persons living with PD were used for the analysis. Datasets were each obtained from single independent studies (Finnish, European, Canadian, Fareo Islands, Swedish, Australian, and Norwegian populations) or combined from two independent studies (American and Caucasian populations). We estimated the proportion of fPD patients in the world population with a formula (% fPD = fPD population/total PD population).

Data from Fig. 2b was obtained from epidemiological studies were identified through Google Scholar search utilizing keywords “Parkinson”, “genetic”, “population”, “familial”, and the relevant gene names (i.e. LRRK2, PRKN/PARK2, ATP13A2, SNCA, VPS35, PINK1, DJ-1, and DNAJC6). A total of 50 epidemiological studies^93–129^ were used for the analysis (total *n* = 488 patients carrying various mutations and 32,012 PD cases). The frequency of mutation (PD population with mutation/total PD population) was averaged across studies examining the same gene. Only studies containing sample sizes of >100 subjects were included in the analysis.

### Meta-analysis of genetic and GWAS studies

A meta-analysis of genetic and GWAS studies is displayed in Fig. 2c. Raw GWAS data were extracted 24 independent genetic studies^8,10–14,130–148^ (identified through Google scholar search with gene name and keywords “Parkinson’s”, “variant”, and “odds ratio”) combined with GWAS database (NHGRI-EBI Catalog, accessed 14th Jan 2019, and filtered for “Parkinson’s” as phenotype trait^149^). The collated data set consisted of 25,243 PD cases and 41,945 healthy controls. The frequency (%) of each variant was measured in 25,243 total PD cases. The odds ratio (OR) was calculated by dividing the frequency of the variant among patients by the frequency of the variant in healthy controls. Only variants with OR greater than 1.5 were plotted against the frequency.

### Data extraction from PDgene GWAS database

GWAS data were extracted from PDgene database^141^ was accessed on 18th Jan 2019 (*n* = 19,061 PD cases and healthy controls). Datasets for the genes listed under “Top Results” were filtered for *p* < 5 × 10^–8^ and OR values were plotted on Fig. 2d.

### Literature search of and meta-analysis of iPSC-PD studies

Original research papers were identified through Google Scholar search utilizing keywords “Parkinson”, “iPSC”, “induced pluripotency stem cell”, “patient-derived”, and “models”. Papers published between 2011 and 2020 which included phenotypic and/multimodal analysis of the patient-derived PD lines were selected for analysis (*n* = 67^24–92^). The meta-analyses of these studies were displayed in Fig. 3b–j.

In Fig. 3j, we meta-analyzed immunocytochemistry quantifications from 33 independent iPSC-PD studies. Studies reporting TUJ1 and TUBBIII were used for calculating the percentages of neurons, both label the bIII-Tubulin protein, and the names are used interchangeably. The percentage of neurons (bIII-Tub/DAPI) was reported by 23 studies (including two of them not reporting trajectories). Percentage of TH neurons (TH+/DAPI) is averaged for 32 studies. However, 12 of these 32 studies did not directly report the percentage of TH+/DAPI but instead reported TH+/TUJ1 and TUJ1+/DAPI. In this instance, for consistency in our analysis, we estimated the proportion of TH+/DAPI with a formula (TH/DAPI = TUJ1/DAPI × TH/TUJ1).

## Supplementary information

nr-reporting-summary
